# Low-dose lithium supplementation promotes adipose tissue browning and sarco(endo)plasmic reticulum Ca^2+^ ATPase uncoupling in muscle

**DOI:** 10.1016/j.jbc.2022.102568

**Published:** 2022-10-07

**Authors:** Mia S. Geromella, Chantal R. Ryan, Jessica L. Braun, Michael S. Finch, Lucas A. Maddalena, Olivia Bagshaw, Briana L. Hockey, Fereshteh Moradi, Rachel K. Fenech, Jisook Ryoo, Daniel M. Marko, Roopan Dhaliwal, Jake Sweezey-Munroe, Sophie I. Hamstra, Georgina Gardner, Sebastian Silvera, Rene Vandenboom, Brian D. Roy, Jeffrey A. Stuart, Rebecca E.K. MacPherson, Val A. Fajardo

**Affiliations:** 1 Department of Kinesiology, Brock University, St. Catharines, Ontario, Canada; 2 Centre for Bone and Muscle Health, Brock University, St. Catharines, Ontario, Canada; 3 Department of Health Sciences, Brock University, St. Catharines, Ontario, Canada; 4 Department of Biological Sciences, Brock University, St. Catharines, Ontario, Canada

**Keywords:** SERCA, sarcolipin, neuronatin, UCP1, beige, uncoupling, efficiency, BAT, brown adipose tissue, DMEM, Dulbecco's modified Eagle's medium, GSK3, glycogen synthase kinase 3, GTT, glucose tolerance testing, HFD, high-fat diet, HSA, human skeletal actin, ITT, insulin tolerance testing, iWAT, inguinal white adipose tissue, KD, knockdown, LiCl, lithium chloride, NNAT, neuronatin, PGC-1α, peroxisome proliferator–activated receptor-gamma coactivator 1-alpha, RYR, ryanodine receptor, SERCA, sarco(endo)plasmic reticulum Ca^2+^-ATPase, SLN, sarcolipin, SR, sarcoplasmic reticulum, UCP1, uncoupling protein 1

## Abstract

Sarco(endo)plasmic reticulum Ca^2+^-ATPase (SERCA) uncoupling in skeletal muscle and mitochondrial uncoupling *via* uncoupling protein 1 (UCP1) in brown/beige adipose tissue are two mechanisms implicated in energy expenditure. Here, we investigated the effects of glycogen synthase kinase 3 (GSK3) inhibition *via* lithium chloride (LiCl) treatment on SERCA uncoupling in skeletal muscle and UCP1 expression in adipose. C2C12 and 3T3-L1 cells treated with LiCl had increased SERCA uncoupling and UCP1 protein levels, respectively, ultimately raising cellular respiration; however, this was only observed when LiCl treatment occurred throughout differentiation. *In vivo*, LiCl treatment (10 mg/kg/day) increased food intake in chow-fed diet and high-fat diet (HFD; 60% kcal)–fed male mice without increasing body mass—a result attributed to elevated daily energy expenditure. In soleus muscle, we determined that LiCl treatment promoted SERCA uncoupling *via* increased expression of SERCA uncouplers, sarcolipin and/or neuronatin, under chow-fed and HFD-fed conditions. We attribute these effects to the GSK3 inhibition observed with LiCl treatment as partial muscle-specific GSK3 knockdown produced similar effects. In adipose, LiCl treatment inhibited GSK3 in inguinal white adipose tissue (iWAT) but not in brown adipose tissue under chow-fed conditions, which led to an increase in UCP1 in iWAT and a beiging-like effect with a multilocular phenotype. We did not observe this beiging-like effect and increase in UCP1 in mice fed a HFD, as LiCl could not overcome the ensuing overactivation of GSK3. Nonetheless, our study establishes novel regulatory links between GSK3 and SERCA uncoupling in muscle and GSK3 and UCP1 and beiging in iWAT.

Adaptive thermogenesis is the cellular process where, in response to prolonged cold exposure or caloric excess, energy expenditure and heat production are increased resulting in a greater combustion of metabolic substrates (1). In mammals, brown adipose tissue (BAT)/beige adipose tissue and skeletal muscle are the primary sites for adaptive thermogenesis. Beige adipose tissue and BAT are characterized by an abundance of mitochondria and high uncoupling protein 1 (UCP1) content, which acts to uncouple the proton gradient from ATP synthesis, ultimately dissipating the stored energy in the form of heat. In skeletal muscle, the sarco(endo)plasmic reticulum Ca^2+^-ATPase (SERCA) pump is a major energy (ATP) consumer and is a known mediator of muscle-based thermogenesis (2, 3). Specifically, the SERCA pump catalyzes the active transport of Ca^2+^ from the cytosol to the sarcoplasmic reticulum (SR), a process that is important for muscle relaxation. Based on its structure, SERCA has two Ca^2+^-binding sites and one ATP-binding site (4), which suggests that under optimal conditions, SERCA can transport two Ca^2+^ ions for every one ATP hydrolyzed (5). Although *in vivo*, the presence of SERCA uncouplers such as sarcolipin (SLN) and the newly identified neuronatin (NNAT), as well as changes in membrane lipid composition or ryanodine receptor (RYR) Ca^2+^ leak, makes the SERCA pump much less efficient, thereby lowering the apparent coupling ratio and increasing energy expenditure and heat release (6, 7, 8, 9, 10).

Both adipose-based (*via* UCP1) and muscle-based (*via* SERCA uncoupling) thermogenesis have shown promise in the fight against obesity—a metabolic disorder caused by a chronic imbalance between excessive caloric intake and a deficit in energy expenditure (8, 9, 11, 12, 13, 14, 15, 16). Thus, discovering novel cellular targets that could promote both adipose-based and muscle-based thermogenesis would likely aid in offsetting the onset or progression of obesity. First recognized for its role in the regulation of glycogen synthase (17), glycogen synthase kinase 3 (GSK3) has been recently identified as a significant contributor to numerous disease states including obesity and diabetes (17). GSK3 is a constitutively active serine/threonine kinase, which exists in two isoforms: GSK3α and GSK3β, though the latter is most dominant in adipose and muscle tissues (17, 18). It has been suggested that the overactivation of GSK3 may contribute to the onset of obesity. For example, GSK3β overexpression in mice resulted in increased body mass and adiposity along with impaired glucose tolerance and insulin sensitivity (19). Mechanistically, the association between GSK3 activity and obesity may in part be mediated through adaptive thermogenesis. Recently, it has been demonstrated that GSK3 negatively regulates BAT–based thermogenesis, where the expression of thermogenic genes, including UCP1, are suppressed by GSK3 activity (18). However, analysis was restricted to the effects of GSK3 on brown adipocytes, and the role of GSK3 on regulating browning of white adipose tissue (WAT) remains unknown. Furthermore, the role of GSK3 in regulating muscle-based thermogenesis and SERCA uncoupling remains unknown. This is important as it was muscle-specific overexpression of GSK3β in mice that resulted in increased adiposity, even under chow-fed conditions (19).

Lithium is a well-known GSK3 inhibitor that has been commonly used in the treatment of bipolar disorder (20, 21). Although higher doses (*i.e.*, ≥1.0 mM serum concentration) taken over a prolonged period have been associated with weight gain and obesity (22), low-dose lithium supplementation produces the opposite effect (23). We have recently shown that trace levels of lithium found naturally in water negatively correlates with the prevalence of obesity (24). In mice, others have shown that low-dose lithium supplementation (provided as lithium chloride [LiCl], 10 mg/kg/day) for 14 weeks attenuated high-fat diet (HFD)–induced obesity and atherosclerosis (23). In skeletal muscle specifically, we have shown that male mice fed this same dose for 6 weeks have reduced GSK3 activation, leading to increased calcineurin activation, peroxisome proliferator–activated receptor-gamma coactivator 1-alpha (PGC-1α) content and fatigue resistance (25). Calcineurin is a Ca^2+^-dependent phosphatase that promotes PGC-1α expression and fatigue resistance (26, 27). Furthermore, recent studies have shown that calcineurin stimulates both muscle- and adipose-based thermogenesis by increasing SLN and UCP1 in muscle and adipose tissue, respectively (13). In muscle, calcineurin is well known to be counteracted by GSK3 (28, 29). Thus, in the present study, we used a cell and rodent model approach to determine whether GSK3 inhibition *via* LiCl supplementation would enhance muscle-based and adipose-based thermogenic mechanisms: SERCA uncoupling and UCP1 expression.

## Results

### Lithium inhibits GSK3 and lowers the transport efficiency of SERCA

**Figure 1:**
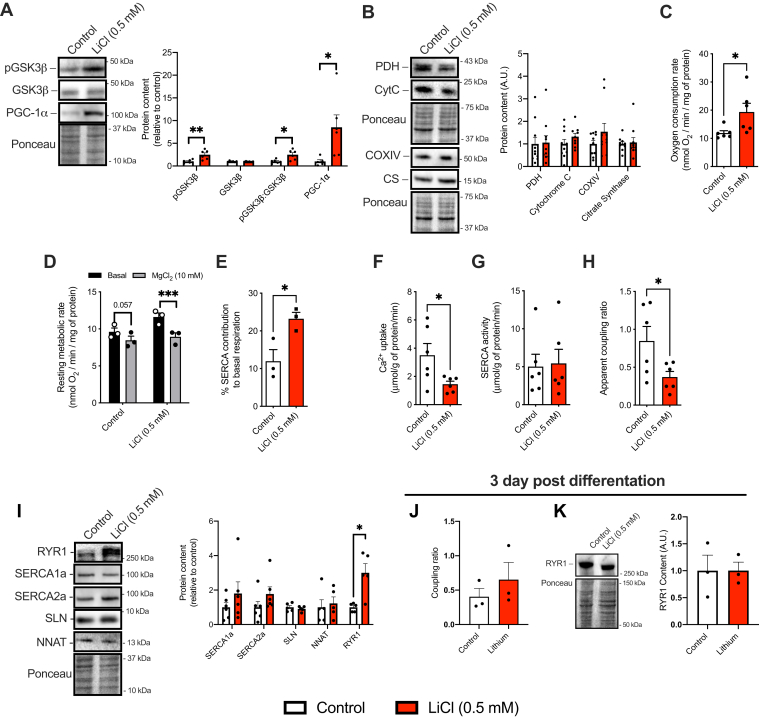
**LiCl treatment inhibits GSK3, increases cellular respiration, and promotes SERCA uncoupling in C2C12 myocytes.***A*, increased inhibitory serine9 phosphorylation of GSK3β and PGC-1α protein with 0.5 mM LiCl treatment (n = 5–6 per group). *B*, no changes in mitochondrial proteins: pyruvate dehydrogenase (PDH), cytochrome *c*, cytochrome *c* oxidase subunit IV (COXIV), or citrate synthase was found with 0.5 mM LiCl treatment (n = 3 per group). *C*, resting cellular respiration rates in LiCl-treated and control C2C12 cells (n = 6 per group). Cellular respiration in the presence and absence of 10 mM MgCl_2_ (*D*) to calculate the energetic contribution of SERCA (*E*) (n = 3 per group). *F*, Ca^2+^ uptake is significantly reduced without altering SERCA activity (*G*), leading to a significant reduction in the apparent coupling ratio of SERCA (*H*) (n = 6 per group). *I*, Western blot analyses of SERCA isoform, SLN, NNAT, and RYR1 in LiCl-treated and control C2C12 cells (n = 5–6 per group). *J* and *K*, acute (3 days) of 0.5 mM LiCl treatment in 10-day differentiated C2C12 cells did not alter SERCA coupling ratio or RYR1 content (n = 3 per group). ∗*p* < 0.05, ∗∗*p* < 0.01 using a Student’s *t* test, with each n representing a technical replicate. All Western blot data are presented as relative to control. All values are means ± SEM. GSK3, glycogen synthase kinase 3; LiCl, lithium chloride; NNAT, neuronatin; PGC-1α, peroxisome proliferator–activated receptor-gamma coactivator 1-alpha; RYR1, ryanodine receptor 1; SERCA, sarco(endo)plasmic reticulum Ca^2+^-ATPase; SLN, sarcolipin.

First, we questioned whether 0.5 mM LiCl would alter SERCA coupling ratio in C2C12 myoblasts, which is a dose that we have previously shown to inhibit GSK3 in this cell line (30). Cells were treated with or without LiCl for 7 days during differentiation. This resulted in a significant increase in inhibitory serine9 GSK3 phosphorylation (Fig. 1*A*). PGC-1α is also negatively regulated by GSK3 (25, 31, 32, 33), and LiCl increased PGC-1α protein content nearly four-fold (Fig. 1*A*); however, this occurred without any significant changes in other mitochondrial markers (Fig. 1*B*). Nonetheless, LiCl-treated C2C12 cells had elevated cellular respiration rates (Fig. 1*C*), and 10 mM MgCl_2_ lowered respiration rates in control and LiCl-treated cells (Fig. 1*D*). This reduction is presumed to have been caused by an inhibition of RYR Ca^2+^ leak that minimizes SERCA activity (3). Calculating the energetic contribution of SERCA as the difference in respiration with and without 10 mM MgCl_2_ revealed a near two-fold increase with LiCl treatment (Fig. 1*E*). Corresponding well with these data, we observed a significant reduction in Ca^2+^ uptake with no change in SERCA activity, leading to a significant reduction in the apparent coupling ratio of SERCA with LiCl treatment (Fig. 1, *F*–*H*). This suggests that the increase in the energy consumption of SERCA is due to a reduction in Ca^2+^ transport efficiency. To investigate the cellular mechanisms leading to this reduction, we examined the protein levels of SERCA1a/2a, SLN, NNAT, and RYR1. No changes were detected in either SERCA isoform, SLN, or NNAT; however, we did observe a significant upregulation in RYR1 (Fig. 1*I*). Thus, the increase in SERCA energy expenditure resulting from a reduction in the apparent coupling ratio of SERCA may be due to enhanced RYR Ca^2+^ leak in LiCl-treated C2C12 cells.

We have previously shown that LiCl treatment enhances myoblast differentiation (30), which could influence our findings. Therefore, we next examined the acute effects of LiCl in 10-day differentiated myoblasts. Our results show that 3 days of LiCl treatment after C2C12 cells were already differentiated produced no effect on coupling ratio or RYR content (Fig. 1, *J* and *K*). This suggests that the effect of LiCl on lowering SERCA efficiency, presumably through increased RYR Ca^2+^ leak, only manifests when the treatment occurs throughout differentiation.

### Lithium inhibits GSK3 and increases UCP1 content in 3T3-L1 preadipocytes

**Figure 2:**
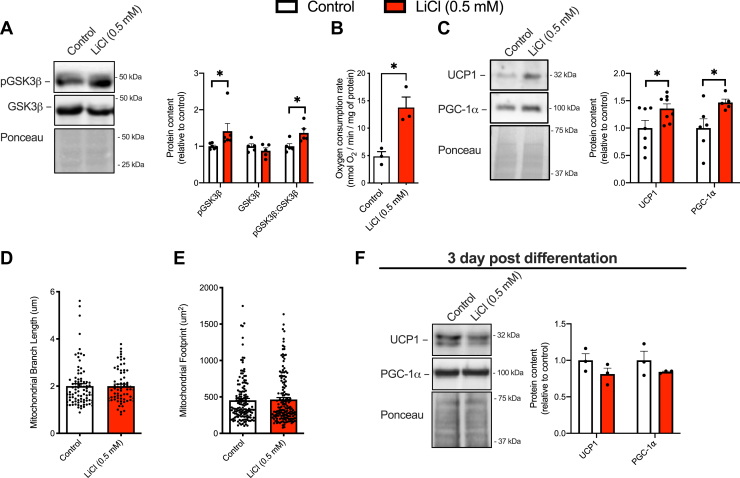
**LiCl treatment inhibits GSK3 and increases UCP1 and cellular respiration in 3T3-L1 adipocytes.***A*, increased inhibitory serine9 phosphorylation of GSK3β with 0.5 mM LiCl treatment (n = 5–6 per group). *B*, resting cellular respiration rates in LiCl-treated and control 3T3-L1 adipocytes (n = 3–4 per group). *C*, UCP1 and PGC-1α protein content measured with Western blotting (n = 6–8 per group). *D* and *E*, mitochondrial footprint and branch length in 3T3-L1 adipocytes treated with LiCl. *F*, acute (3 days) of 0.5 mM LiCl treatment in 10-day differentiated 3T3-L1 adipocytes did not alter UCP1 or PGC-1α protein content (n = 3 per group). ∗*p* < 0.05 using a Student’s *t* test, with each n representing a technical replicate. All Western blot data are presented as relative to control. All values are means ± SEM. GSK3, glycogen synthase kinase 3; LiCl, lithium chloride; PGC-1α, peroxisome proliferator–activated receptor-gamma coactivator 1-alpha; UCP1, uncoupling protein 1.

We next examined the effects of LiCl treatment on UCP1 content in 3T3-L1 adipocytes. As with C2C12 cells, 3T3-L1 cells were treated with 0.5 mM LiCl for 10 days during differentiation. As expected, there was a significant increase in inhibitory serine9 phosphorylation of GSK3β with LiCl treatment compared with nontreated cells (Fig. 2*A*). This inhibition was associated with an increase in cellular respiration (Fig. 2*B*) and increased UCP1 and PGC-1α content (Fig. 2*C*), further supporting the role of GSK3 in negatively regulating UCP1 expression in adipocytes. However, LiCl treatment had no impact on the mitochondrial footprint, indicating no increase in mitochondrial abundance (Fig. 2, *D* and *E*).

The acute effects of LiCl treatment were also investigated in differentiated 3T3-L1 adipocytes. As observed previously with C2C12 myocytes, 3 days of LiCl treatment did not alter the protein levels of neither UCP1 nor PGC-1α (Fig. 2*F*). This again indicates that the ability of LiCl to affect UCP1 and PGC-1α levels requires that the treatment occurs during differentiation.

### Low-dose lithium supplementation in chow-fed and HFD-fed mice

**Figure 3:**
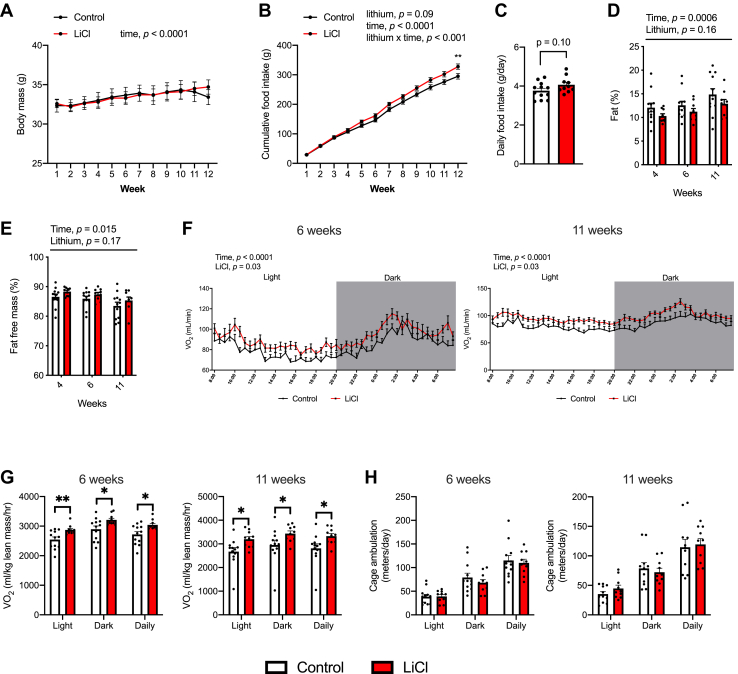
**LiCl supplementation does not alter body mass despite increases in food intake because of increases in daily energy expenditure in male C57BL/6J chow-fed mice.***A*, weekly analysis of body mass throughout the 12-week LiCl treatment period (n = 10–12 per group). *B*, cumulative food intake throughout the 12-week LiCl treatment period (n = 10–12 per group). *C*, daily food intake in control and LiCl-fed mice (n = 10–12 per group). *D* and *E*, body fat and fat-free mass measured through small animal DXA (n = 10–12 per group). *F*, absolute energy expenditure (VO_2_) in control and LiCl-treated mice at 6 and 11 weeks of treatment (n = 10–12 per group). *G*, relative energy expenditure (per lean mass) in control and LiCl-treated mice at 6 and 11 weeks of treatment (n = 10–12 per group). *H*, cage activity in control and LiCl-treated mice (n = 10–12 per group). For *A*, *B*, *D*–*F*, a two-way repeated-measures ANOVA was used to test the main effects of time, lithium, and their potential interaction. For *C* and *G*, comparisons between control and LiCl were made using independent Student's *t* tests, ∗*p* < 0.05. All values are means ± SEM. DXA, dual-energy X-ray absorbtiometry; LiCl, lithium chloride.

**Figure 4:**
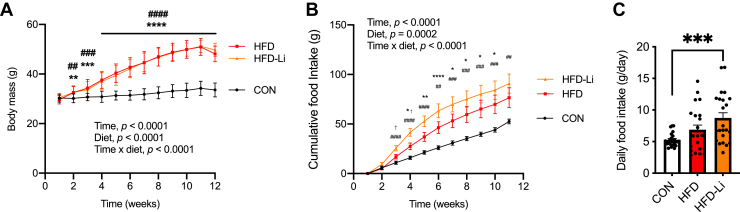
**LiCl supplementation does not alter body mass despite increases in food intake in male C57BL/6J high-fat diet (HFD)–fed mice.***A*, weekly analysis of body mass throughout the 12-week LiCl treatment period (n = 24 per group). *B*, cumulative food intake throughout the 12-week LiCl treatment period (n = 24 per group). *C*, daily food intake in control and LiCl-fed mice (n = 24 per group). For *A* and *B*, two-way ANOVA was used to test the main effects of time, diet, and their potential interaction. ∗∗*p* < 0.01, ∗∗∗*p* < 0.001, and ∗∗∗∗*p* < 0.0001 for HFD *versus* control (CON, chow); ^#^*p* < 0.05, ^##^*p* < 0.01, ^###^*p* < 0.001, ^####^*p* < 0.0001 for HFD-Li *versus* control; ^†^*p* < 0.05 for HFD *versus* HFD-Li. For *C*, a one-way ANOVA with a Tukey’s post hoc test was used, ∗∗∗*p* < 0.001. All values are means ± SEM. LiCl, lithium chloride.

To determine if LiCl could enhance muscle-based and adipose-based thermogenesis *in vivo*, we treated chow-fed and HFD-fed mice with a dose we had previously shown to cause GSK3 inhibition in muscle (25). Under a chow diet, 12 weeks of LiCl supplementation (10 mg/kg/day *via* drinking water) did not alter body mass; however, LiCl treatment appeared to increase food consumption with a significant difference in cumulative food intake by week 12 compared with control (Fig. 3, *A* and *B*). Daily food intake in the LiCl group also tended to be higher compared with control, but this was not statistically significant (Fig. 3*C*). Furthermore, body composition analysis showed no significant differences between LiCl and control (Fig. 3, *D* and *E*). This apparent increase in food consumption without an increase in body mass or percent of body fat points toward an increase in energy expenditure with LiCl supplementation, which was observed across light, dark, and daily periods (Fig. 3, *F* and *G*). In both absolute and relative to lean mass, O_2_ consumption was significantly elevated in the LiCl group at both 6 weeks and 11 weeks of treatment. Importantly, these changes in energy expenditure could not be explained by any differences in cage ambulation (Fig. 3*H*). Similar findings were also observed in mice fed an HFD (60% kcal from fat), a condition that would benefit from adaptive thermogenesis. Like the chow-fed mice, no differences in body mass between HFD and HFD-Li groups were observed after 12 weeks of feeding (Fig. 4*A*) despite the HFD-Li group having the highest levels of cumulative food consumption and daily food intake (Fig. 4, *B* and *C*). We presume this to be due to elevated daily energy expenditure, similar to what was found in the chow study (Fig. 3). However, at the time of this experiment, we did not have the Promethion Metabolic Cages nor Small Animal DXA scanner in place to conduct measures of O_2_ consumption or body composition.

### LiCl supplementation promotes SERCA uncoupling in chow-fed and HFD-fed mice

**Figure 5:**
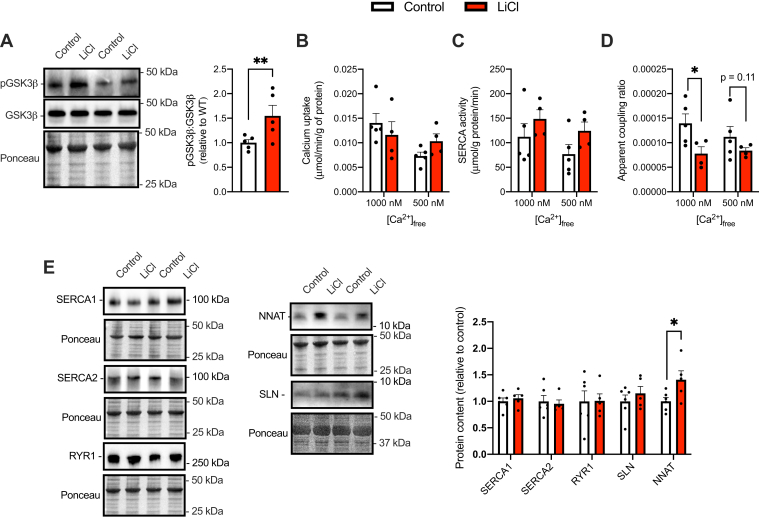
**LiCl supplementation inhibits GSK3 and promotes SERCA uncoupling in soleus muscles from male C57BL/6J chow-fed mice.***A*, inhibitory serine9 phosphorylation of GSK3β in soleus muscles from control and LiCl-treated mice (n = 5 per group). *B*, rates of Ca^2+^ uptake in soleus muscles from control and LiCl-treated mice (n = 5 per group). *C*, SERCA activity in soleus muscles from control and LiCl-treated mice (n = 5 per group). *D*, apparent coupling ratio (Ca^2+^ uptake divided by SERCA activity at matching [Ca^2+^]_free_) (n = 5 per group). *E*, Western blot analysis of SERCA1, SERCA2, RYR1, SLN, and NNAT. For SERCA2 and RYR1, these targets were probed for on the same membrane and have therefore the same Ponceau. ∗*p* < 0.05, ∗∗*p* < 0.01, using an independent Student’s *t* test. All values are means ± SEM. GSK3, glycogen synthase kinase 3; LiCl, lithium chloride; NNAT, neuronatin; RYR, ryanodine receptor; SERCA, sarco(endo)plasmic reticulum Ca^2+^-ATPase.

SERCA coupling ratio was tested in the soleus muscle from LiCl-supplemented and control mice. This muscle was chosen based on its known expression of SERCA uncouplers, SLN and NNAT (6, 10, 34). Under a chow diet, western blot analysis showed a significant increase in inhibitory serine9 phosphorylation on GSK3β in soleus muscles from LiCl-treated mice *versus* control (Fig. 5*A*). When measuring rates of Ca^2+^ uptake and SERCA activity separately, we did not find any significant effect of LiCl treatment; however, LiCl treatment resulted in a significant reduction in the apparent coupling ratio, particularly at 1000 nM [Ca^2+^]_free_ (Fig. 5, *B*–*D*). The promotion of SERCA uncoupling was associated with a significant increase in NNAT but not SLN (Fig. 5*E*). We also did not observe any changes in SERCA or RYR1 content (Fig. 5*E*).

**Figure 6:**
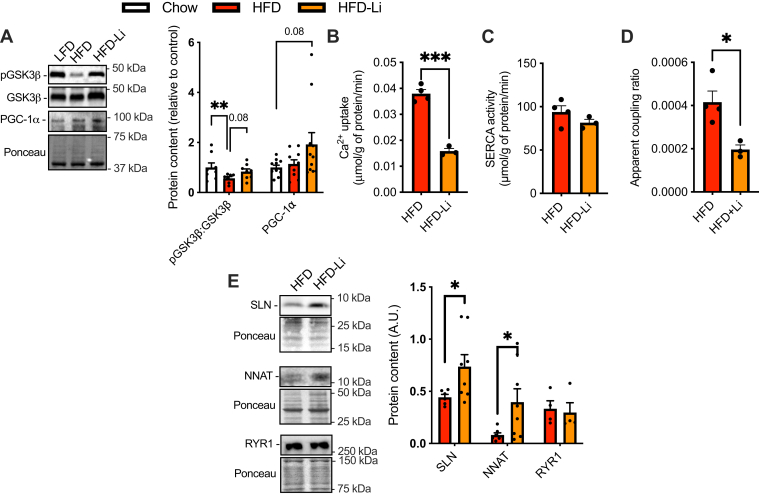
**LiCl supplementation inhibits GSK3 and promotes SERCA uncoupling in soleus muscles from male C57BL/6J high-fat diet (HFD)–fed mice.***A*, inhibitory serine9 phosphorylation of GSK3 and PGC-1α protein in soleus muscles from control (CON; standard chow) HFD and HFD-Li mice (n = 8–11 per group). *B*, rates of Ca^2+^ uptake in soleus muscles obtained from HFD and HFD-Li mice (n = 3–4 per group with each n representing pooled soleus from three mice). *C*, SERCA activity in soleus muscles obtained from HFD and HFD-Li mice (n = 3–4 per group with each n representing pooled soleus from three mice). *D*, apparent coupling ratio (Ca^2+^ uptake divided by SERCA activity at matching [Ca^2+^]_free_). *E*, Western blot analyses of SERCA uncouplers SLN and NNAT as well as RYR1 (n = 6–8 per group). For *A*, ∗∗*p* < 0.01 with a one-way ANOVA and Tukey’s post hoc test. For *B*–*E*, ∗*p* < 0.05, ∗∗∗*p* < 0.001 with a Student’s *t* test. All values are means ± SEM. GSK3, glycogen synthase kinase 3; LiCl, lithium chloride; NNAT, neuronatin; PGC-1α, peroxisome proliferator–activated receptor-gamma coactivator 1-alpha; RYR, ryanodine receptor; SERCA, sarco(endo)plasmic reticulum Ca^2+^-ATPase; SLN, sarcolipin.

Western blot analysis revealed that the consumption of an HFD significantly reduced GSK3β serine9 phosphorylation, although this effect was blunted with LiCl treatment (Fig. 6*A*). Furthermore, PGC-1α content appeared to be elevated in HFD-Li supplemented mice in comparison to chow-fed controls; however, this was not significantly different (Fig. 6*B*). With respect to SERCA, we identified a significant reduction in Ca^2+^ uptake in the HFD-Li group when compared with untreated counterparts, whereas, there were no differences between groups in SERCA activity (Fig. 6, *B* and *C*). We also found a significant reduction in the apparent coupling ratio of SERCA in the soleus of HFD-Li group compared with HFD (Fig. 6*D*). Notably, the rates of Ca^2+^ uptake, activity, and the calculated apparent coupling ratio were obtained at a 1500 nM [Ca^2+^]_free_ since Ca^2+^ uptake in soleus muscles from HFD-fed mice (with or without LiCl) did not reach levels below 1000 nM [Ca^2+^]_free_. To identify the cellular mechanisms leading to the reduction in apparent coupling ratio observed with a HFD, we examined the protein levels of SERCA uncoupling proteins SLN and NNAT as well as RYR1. Our results show a significant increase in SLN and NNAT content in HFD-Li *versus* HFD soleus; however, there were no differences in RYR1 (Fig. 6*E*).

**Figure 7:**
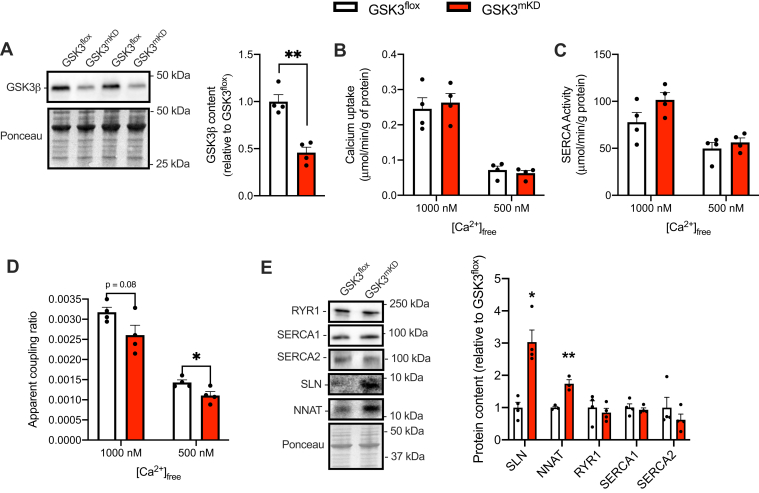
**Muscle-specific partial GSK3 knockdown (GSK3**^**mKD**^**) promotes SERCA uncoupling in soleus muscles from chow-fed male C57BL/6J mice.***A*, GSK3β protein content assessed *via* Western blotting in soleus muscles from GSK3 floxed (GSK3^floxed^, control) and GSK3^mKD^ mice (n = 4 per group). *B*, rates of Ca^2+^ uptake in soleus muscles from GSK3^floxed^ and GSK3^mKD^ mice (n = 4 per group). *C*, SERCA activity in soleus muscles from GSK3^floxed^ and GSK3^mKD^ mice (n = 4 per group). *D*, apparent coupling ratio (Ca^2+^ uptake divided by SERCA activity at matching [Ca^2+^]_free_) (n = 4 per group). *E*, Western blot analysis of SERCA1, SERCA2, RYR1, SLN, and NNAT (n = 3–4 per group). ∗*p* < 0.05, ∗∗*p* < 0.01, using an independent Student’s *t* test. All values are means ± SEM. GSK3, glycogen synthase kinase 3; NNAT, neuronatin; RYR, ryanodine receptor; SERCA, sarco(endo)plasmic reticulum Ca^2+^-ATPase; SLN, sarcolipin.

Acknowledging the fact that Li also has GSK3-independent effects, we generated a partial muscle-specific GSK3 knockdown (GSK3^mKD^) mouse colony. These mice heterozygously express the flox sequence flanking both GSK3 α and β, whereas also heterozygously expressing human skeletal actin (HSA)-Cre to direct skeletal muscle–specific KD of GSK3. In turn, the GSK3^mKD^ mice display a ∼55% reduction in GSK3β in the soleus compared with GSK3^floxed^ control mice (Fig. 7*A*). Similar to LiCl treatment and under a chow diet, we did not find any differences in rates of Ca^2+^ uptake or SERCA activity when examined separately; however, a significant reduction in the apparent coupling ratio of SERCA, particularly at 500 nM of [Ca^2+^]_free_, was detected (Fig. 7, *B*–*D*). Moreover, while we did not observe any differences in RYR1 content, both SLN and NNAT were elevated in muscles from GSK3^mKD^ mice compared with GSK3^floxed^ mice (Fig. 7*E*).

### LiCl supplementation promotes iWAT beiging in chow-fed but not HFD-fed mice

**Figure 8:**
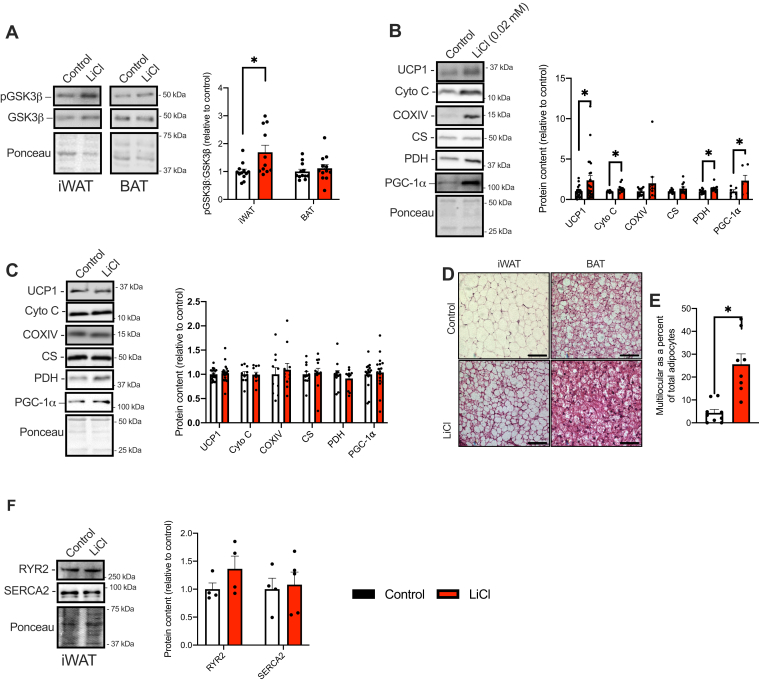
**LiCl supplementation inhibits GSK3 and promotes a beige-like phenotype in iWAT from mice fed a chow diet.***A*, inhibitory serine9 GSK3 phosphorylation is elevated in iWAT but not BAT after LiCl treatment (n = 11–12 per group). *B* and *C*, Western blot analyses of mitochondrial proteins UCP1, cytochrome *c* (Cyto C), cytochrome *c* oxidase subunit IV (COXIV), citrate synthase (CS), pyruvate dehydrogenase E1-alpha subunit (PDH), and PGC-1α in iWAT (*B*) and BAT (*C*) from control and LiCl-fed mice (n = 6–12 per group, apart from UCP1 where 18 per group were used). *D*, H&E stain of iWAT and BAT sections from control and LiCl-fed mice (the scale bar represents 100 μm). *E*, percent of multilocular adipocytes quantified using ImageJ. *F*, Western blot quantification of SERCA2 and RYR2 in iWAT (n = 4 per group). ∗*p* < 0.05 using a Student’s *t* test. All Western blot data are presented as relative to control. All values are means ± SEM. BAT, brown adipose tissue; GSK3, glycogen synthase kinase 3; iWAT, inguinal white adipose tissue; LiCl, lithium chloride; PGC-1α, peroxisome proliferator–activated receptor-gamma coactivator 1-alpha; RYR, ryanodine receptor; SERCA, sarco(endo)plasmic reticulum Ca^2+^-ATPase; UCP1, uncoupling protein 1.

We next examined the effects of LiCl treatment on both iWAT and BAT in chow-fed and HFD-fed mice. In chow-fed mice and as early as 6 weeks of treatment, LiCl significantly increased GSK3 serine9 phosphorylation in iWAT (Fig. 8*A*); however, this was not observed in BAT (Fig. 8*A*). In turn, LiCl treatment resulted in significant elevations of UCP1 and several other mitochondria-related proteins including PGC-1α in iWAT but not BAT (Fig. 8, *B* and *C*). Histological analysis demonstrated for the first time that GSK3 inhibition associated with LiCl supplementation leads to a significant “beiging” effect on iWAT, taking on a multilocular-like phenotype (Fig. 8, *D* and *E*). As SERCA-mediated Ca^2+^ signaling has been shown to contribute to energy homeostasis in beige adipocytes (35), we next examined whether LiCl supplementation would alter protein levels of SERCA2 and RYR2 in iWAT. However, we did not find any differences in either SERCA2 or RYR2 content (Fig. 8*F*).

**Figure 9:**
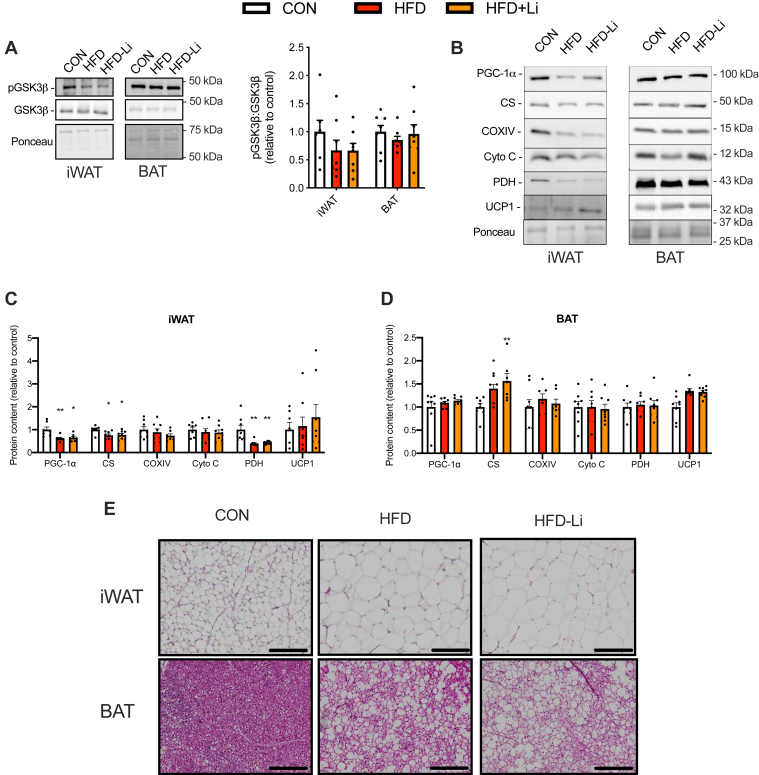
**LiCl supplementation did not inhibit GSK3 in iWAT or BAT from mice fed a high-fat diet (HFD).***A*, inhibitory serine9 phosphorylation of GSK3 in iWAT and BAT from control (CON; standard chow) HFD and HFD-Li mice (n = 8 per group). *B*–*D*, representative Western blot images and analyses of mitochondrial proteins PGC-1α, citrate synthase (CS), cytochrome *c* oxidase subunit IV (COXIV), pyruvate dehydrogenase E1-alpha subunit (PDH), and UCP1 in iWAT (*C*) and BAT (*D*). *E*, H&E stain of iWAT and BAT sections from chow, HFD, and HFD-Li mice (the scale bar represents 200 μm). For *B* and *C*, ∗*p* < 005, ∗∗*p* < 0.01 with a one-way ANOVA and Tukey’s post hoc test. Western blot data are presented as relative to chow. All values are means ± SEM. BAT, brown adipose tissue; GSK3, glycogen synthase kinase 3; iWAT, inguinal white adipose tissue; LiCl, lithium chloride; PGC-1α, peroxisome proliferator–activated receptor-gamma coactivator 1-alpha; UCP1, uncoupling protein 1.

In contrast with chow-fed mice, LiCl supplementation did not lead to any alterations in iWAT or BAT in HFD-fed mice (Fig. 9). There were no differences in inhibitory serine9 phosphorylation of GSK3β between HFD and HFD-Li groups when expressed as a ratio of total GSK3β (Fig. 9*A*). When assessed separately, our results show that the HFD led to a significant ∼44% reduction in serine9 phosphorylated GSK3β in both the HFD (*p* = 0.01) and HFD-Li (*p* = 0.01) groups compared with control (*F* = 5.8, *p* = 0.009). This occurred without any changes in total GSK3β (*F* = 0.14, *p* = 0.86). This suggests that LiCl supplementation could not overcome the overactivation of GSK3 in iWAT observed with high fat feeding. When measuring protein content in iWAT, there were significant reductions found in several mitochondrial-related proteins with no change in UCP1 in both HFD and HFD-Li groups compared with chow-fed mice (Fig. 9, *B* and *C*). In BAT, there were no differences observed for UCP1 or most mitochondrial proteins, except for citrate synthase, which was upregulated under both HFD conditions (Fig. 9, *B* and *D*). Finally, histological analyses did not reveal a beige-like phenotype in iWAT from the HFD-Li group (Fig. 9*E*).

### LiCl supplementation does not alter glucose handling in chow-fed or HFD-fed mice

**Figure 10:**
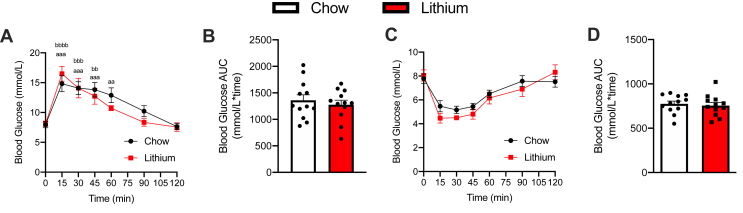
**Glucose tolerance test (GTT) and insulin tolerance test (ITT) in control and LiCl-treated chow-fed mice.***A* and *B*, GTT curves and corresponding area under the curve (AUC) analysis. *C* and *D*, ITT curves and corresponding AUC analysis (n = 12 per group). LiCl, lithium chloride.

**Figure 11:**
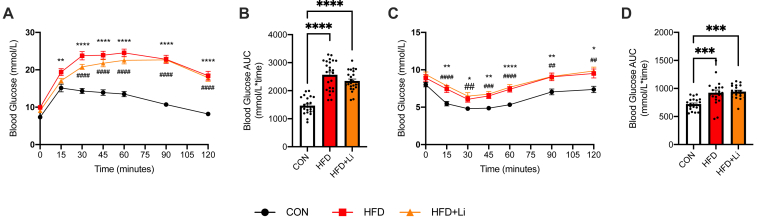
**Glucose tolerance test (GTT) and insulin tolerance test (ITT) in control and LiCl-treated control (CON), high-fat diet (HFD), and HFD-lithium (HFD-Li)-fed mice.***A* and *B*, GTT curves and corresponding area under the curve (AUC) analysis. *C* and *D*, ITT curves and corresponding AUC analysis. For *A* and *C*: ∗*p* < 005, ∗∗*p* < 0.01, ∗∗∗*p* < 0.001, and ∗∗∗∗*p* < 0.0001 for HFD *versus* chow; and ^#^*p* < 005, ^##^*p* < 0.01, ^###^*p* < 0.001, and ^####^*p* < 0.0001 for HFD-Li *versus* chow with a two-way ANOVA and a Tukey’s post hoc test. For *B* and *D*: ∗∗∗*p* < 0.001 and ∗∗∗∗*p* < 0.0001 with a one-way ANOVA and a Tukey’s post hoc test (n = 22–24 per group). LiCl, lithium chloride.

Lithium is known to have insulin-sensitizing effects, and Choi *et al.* (23) found that LiCl treatment, with the same dose used in this study, lowered fasting glucose in HFD-fed mice. Here, we did not find any effect of LiCl supplementation on glucose or insulin tolerance in either chow-fed (Fig. 10) or HFD-fed mice (Fig. 11).

## Discussion

In this study, we examined whether GSK3 inhibition, *via* LiCl supplementation, would promote SERCA uncoupling in C2C12 cells and in skeletal muscles obtained from chow-fed and HFD-fed mice. We also examined whether LiCl supplementation would enhance UCP1 expression in 3T3-L1 adipocytes, and in iWAT and BAT from chow-fed and HFD-fed mice.

Using cellular models first, our results demonstrate that LiCl treatment enhanced respiration in C2C12 cells and 3T3-L1 adipocytes. In C2C12 cells, the increase in respiration was accounted for, at least in part, by an approximately twofold increase in the energetic contribution of SERCA to respiration as estimated with MgCl_2_ experiments that indirectly inhibit SERCA by lowering SR Ca^2+^ leak (3). This increase in the energy expenditure of SERCA was likely because of enhanced SERCA uncoupling, which was mediated through an increase in RYR rather than any changes in known regulatory proteins that can uncouple the SERCA pump. An increase in RYR can lower the transport efficiency of SERCA *via* Ca^2+^ leak as enhanced RYR Ca^2+^ leak is known to cause futile Ca^2+^ cycling with SERCA, thereby increasing energy expenditure and heat release (35, 36, 37, 38). In 3T3-L1 adipocytes, we attribute the increase in energy expenditure to an increase in PGC-1α and UCP1, which was expected based on previous work showing that GSK3 negatively regulated UCP1 expression in BAT (18). However, in both adipocytes and myocytes, the effect of LiCl treatment was abolished when treatment occurred acutely, for 3 days, after cellular differentiation. Together, these results show that in cultured adipocytes and myocytes, LiCl supplementation and GSK3 inhibition throughout cellular differentiation can increase energy expenditure *via* alterations in UCP1 and SERCA Ca^2+^ transport efficiency.

To determine if these effects would translate to the *in vivo* setting, we treated mice with 10 mg/kg/day of LiCl for 12 weeks under chow-fed and HFD-fed conditions. Though we have previously shown that this dose results in a serum concentration that is much lower than that used in our cell culture experiments (0.02 mM *versus* 0.5 mM, respectively) (39, 40, 41), this dose and duration were effective in inhibiting GSK3 and increasing PGC-1α in murine soleus muscle (25). Here, our results show that LiCl supplementation did not alter body mass despite increasing total food intake in both chow-fed and HFD-fed conditions. We attribute this effect to an increase in daily energy expenditure in LiCl-treated mice. However, since this was only measured in chow-fed conditions, we can only assume that this was also the case in HFD-fed mice.

In skeletal muscle, the SERCA pumps account for ∼50% of resting metabolic rate and were estimated to account for 22 to 25% of total daily energy expenditure (3). Thus, the SERCA pumps may be viewed as a metabolic hub in muscle, and alterations in SERCA Ca^2+^ transport efficiency may influence total daily energy expenditure, given the energetic nature of skeletal muscle. In chow-fed mice, the reduction in SERCA Ca^2+^ transport efficiency was observed at 1000 nM [Ca^2+^]_free_; however, statistical significance was lost at 500 nM [Ca^2+^]_free_. Similarly, when fed a HFD, LiCl treatment significantly reduced SERCA coupling ratio, though we could only measure this at 1500 nM [Ca^2+^]_free_ as Ca^2+^ uptake in these samples did not reach levels below 1000 nM. While the exact reason for this remains unknown, we believe that this finding is indicative of an impairment in Ca^2+^ uptake or excessive leak of Ca^2+^ from the SR with a HFD (42). Nonetheless, under both chow-fed and HFD-fed conditions, LiCl treatment significantly reduced the apparent coupling ratio of SERCA, which could contribute at least in part to the increase in daily energy expenditure.

Unlike C2C12 cells, the promotion of SERCA uncoupling *in vivo* did not occur with any changes in RYR content, which highlights an important difference between cell culture and *in vivo* models. Instead, in chow-fed and HFD-fed mice, LiCl treatment lowered SERCA Ca^2+^ transport efficiency by increasing the expression of SERCA uncoupling proteins. In chow-fed mice, LiCl treatment significantly increased NNAT without altering SLN content. This sole increase in NNAT content in chow-fed conditions was sufficient in lowering SERCA Ca^2+^ transport efficiency. Conversely, in HFD-fed mice, both SLN and NNAT were significantly upregulated with LiCl treatment. This is interesting since both SLN (43) and NNAT (10) content were recently shown to be lowered in soleus muscles obtained from mice fed a HFD. It is thus tempting to speculate that the enhancement in GSK3 activation observed here with high fat feeding (and blunted with LiCl treatment) could contribute to the lowered expression of SLN and NNAT content in mice fed a HFD. In further support of this, partial skeletal muscle–specific GSK3 KD increased both SLN and NNAT in soleus muscle, ultimately reducing the apparent coupling ratio of SERCA. While future studies in our laboratory will investigate the effects of high fat feeding in the GSK3^mKD^ mice, the results presented in this study establish a novel regulatory link between GSK3 and the expression of both SLN and NNAT and SERCA Ca^2+^ transport efficiency in skeletal muscle.

In adipose tissue, we questioned whether LiCl treatment could increase UCP1 in iWAT and BAT by inhibiting GSK3, ultimately contributing to changes in daily energy expenditure. Under chow-fed conditions, our results show that LiCl treatment significantly inhibited GSK3 in iWAT, which in turn increased UCP1 and PGC-1α content and promoted a multilocular histological phenotype. To our knowledge, ours is the first study establishing a beiging-like effect of LiCl and GSK3 inhibition in iWAT obtained from mice. However, there was no effect of LiCl on UCP1 or PGC-1α in BAT—a result we attributed to an inability to inhibit GSK3 in this specific adipose depot. Interestingly, when mice were fed a HFD, LiCl treatment did alter neither the UCP1 or PGC-1α content nor the histological appearance of iWAT, which we also attribute to an inability to inhibit GSK3. Similar to our findings in skeletal muscle, GSK3 was more active in iWAT from HFD-fed mice with significantly lowered serine9 phosphorylation. However, unlike skeletal muscle, LiCl treatment could not attenuate this effect. Thus, in combination with our *in vitro* results, these findings demonstrate that when GSK3 is inhibited with LiCl, UCP1 and PGC-1α are increased and may elevate energy expenditure. Future studies from our laboratory will investigate the effects of more potent GSK3 inhibitors and adipose tissue–specific GSK3 KD on iWAT beiging.

The enhancement of GSK3 activation observed in both adipose and muscle obtained from mice fed a HFD may suggest a role for GSK3 overactivation in diet-induced obesity. In this context, previous work conducted by Choi *et al.* (23) found that treating mice with LiCl for 10 mg/kg/day (same dose used in this study) reduced the weight gained after an HFD. Moreover, lithium is known to have insulin-sensitizing effects, and Choi *et al.* found that LiCl treatment in HFD-fed mice lowered fasting glucose levels. However, in our hands, we did not find any alterations in body mass or glucose handling in chow-fed or HFD-fed conditions. It should be noted that Choi *et al.* used an HFD that only comprised 20% fat, whereas we provided a 60% fat diet in our study, which strongly induced obesity in mice and may have overpowered any effect of LiCl. Moreover, the increase in food intake found here in chow-fed and HFD-fed mice treated with LiCl could have masked the effect of LiCl on lowering body mass and potentially glucose handling. Thus, our study is limited in that we did not conduct pair-feeding experiments. Our study is also limited in that we only utilized male mice, primarily to avoid potential confounding effects of hormonal shifts in female mice. However, future studies should examine the effects of LiCl treatment and GSK3 inhibition in female mice.

Nonetheless, our study does provide novel insight into the effect of lithium on appetite and food intake. Lithium is conventionally used in the treatment of bipolar disorder although clinical use of lithium is prescribed at higher doses (serum concentration of 0.5–1.0 mM) in order to overcome the blood–brain barrier and exert beneficial effects on mental health (44). At this high dose, it has been suggested that some patients undergoing lithium therapy are at an increased risk of developing obesity (22), perhaps because of an increase in appetite (45). Here, we observed that low-dose LiCl-supplemented mice also had increased appetite, consuming more food than controls, which is consistent with high-dose lithium therapy. However, at low doses of LiCl, our results show that this increase in appetite and food intake was met with an increase in SERCA uncoupling and/or UCP1 expression, which we believe attenuated any additional weight gain expected with an increase in food intake. Whether the promotion of SERCA uncoupling and UCP1 in skeletal muscle and adipose tissue, respectively, is lost with higher doses of lithium should be investigated with future studies.

In conclusion, our study examined the effects of GSK3 inhibition *via* low-dose LiCl supplementation on SERCA uncoupling in skeletal muscle and UCP1 expression in adipose tissue. Our results provide novel regulatory connections between GSK3 and the expression of NNAT and SLN and SERCA uncoupling in skeletal muscle; and for the first time show that GSK3 inhibition can promote a beiging-like effect in iWAT.

## Experimental procedures

### Cell culture

C2C12 myoblasts were grown in growth medium (Dulbecco's modified Eagle's medium [DMEM-6429] supplemented with 10% fetal bovine serum, 2× nonessential amino acids, 1% penicillin/streptomycin) in a humidified 5% CO_2_ incubator regulated to 5% O_2_ and 37 °C. Upon reaching 80% confluence, C2C12 cells were differentiated using a differentiation medium (DMEM-6429 supplemented with 1% adult horse serum, 1% penicillin–streptomycin, and 2% nonessential amino acids) with and without (control) 0.5 mM LiCl for 7 days, replenishing the media every other day. In a separate set of experiments used to determine the acute effects of LiCl treatment, C2C12 cells were differentiated for 10 days prior to treatment with and without 0.5 mM LiCl for another 3 days. All C2C12 cells were then trypsinized and isolated for further experimentation.

3T3-L1 cells were propagated in DMEM-4629 containing 10% fetal bovine serum, 4 mM glutamine, pyruvate, NaHCO_3_, 2× nonessential amino acids, and 1% penicillin/streptomycin in a humidified 5% CO_2_ incubator regulated to 5% O_2_ and 37 °C. To promote differentiation into adipocyte-like cells, 3T3-L1 cells were cultured as aforementioned in DMEM with the addition of 0.5 mM IBMX, 1 μM dexamethasone, and 10 μg/ml insulin with and without (control) 0.5 mM of LiCl for 10 days, replenishing the media every 72 h. In a separate set of experiments used to determine the acute effects of LiCl treatment, 3T3-L1 cells were differentiated for 10 days prior to treatment with and without 0.5 mM LiCl for another 3 days. Adipocytes were then trypsinized and isolated for further experimentation.

### Respiration

Basal oxygen consumption rates of intact 3T3-L1 and C2C12 cells were measured at 37 °C within a thermostatically controlled closed chamber using a Clark-type oxygen electrode (Rank Brothers Dual Digital Model 20 Respirometer) as previously described (46, 47). Differentiated 3T3-L1 and C2C12 cells (with and without LiCl) were harvested by trypsinization and centrifugation (240*g* for 3 min) prior to being resuspended in 1 ml of differentiation media without LiCl (serving as respiration buffer) and placed into the chamber maintained at 37 °C. In C2C12 cells, oxygen consumption rates were measured in the presence or the absence of 10 mM MgCl_2_, which inhibits Ca^2+^ leak allowing for an indirect measure of the energetic contribution of SERCA (3, 9). Following the recording, the cell suspension was centrifuged (2200*g* for 3 min), and the supernatant was discarded and resuspended in radioimmunoprecipitation assay buffer for protein concentration determination *via* a Bicinchoninic acid assay (Sigma–Aldrich—B9643; VWR—BDH9312).

### Fluorescence microscopy

Fluorescence micrographs of live 3T3-L1 cells were obtained using a Carl Zeiss Axio Observer, Z1 inverted light/epifluorescence microscope equipped with Apotome.2 optical sectioning, and a Hamamatsu ORCA-Flash 4.0V2 digital camera. 3T3-L1 cells were cultured on MatTek 35 mm collagen-coated glass bottom culture dishes as aforementioned. After 10 days of differentiation, media were replaced with DMEM supplemented with 20 nM tetramethyl rhodamine methyl ester and incubated for 30 min to visualize mitochondrial footprint and networks as previously described (48, 49). Cells were viewed with a Plan-Apochromat 63×/1.40 Oil DIC M27 microscope objective. The microscope stage maintained at a humidified 5% CO_2_, 5% O_2_, and 37 °C environment. Red fluorescence was detected using a 540 to 552 nm excitation and 500 to 550 nm emission filter set. Intensity of fluorescence illumination and camera exposure time were held constant throughout the experiment.

### Animals and study design

Experimental protocols were approved by the Brock University Animal Care Committee (file #17-06-03, #19-04-01, and #20-07-01) and were in compliance with the Canadian Council on Animal Care. For the lithium feeding studies, C57BL/6J male mice (16 weeks of age) were ordered from The Jackson Laboratory and allowed to acclimatize for 7 days in the Brock University Comparative Biosciences Facility. During acclimatization, mice were fed standard chow (2014 Teklad global, 14% protein rodent maintenance diet, Harlan Teklad), and after the acclimatization, mice were either fed standard chow or an HFD (60% kcal fat, D12492 rodent HFD, Research Diets). All mice were kept on a 12-h light:12-h dark cycle and had ad libitum access to food and water through the entirety of the study. Mice were housed at a temperature of 22 to 24 °C for the duration of the study.

For the chow-fed study, mice were randomly assigned into one of two experimental groups: (1) control (CON) and (2) low-dose lithium supplementation for 6 to 12 weeks as previously described (25, 39, 40). The low-dose lithium supplementation group received LiCl *via* their drinking water at a dose of 10 mg/kg body weight/day, which results in a serum lithium concentration of 0.02 mM ± 0.001 (39, 40). For the HFD study, mice were randomly assigned into one of three experimental groups: (1) chow, (2) HFD, and (3) HFD-Li where the latter was provided 10 mg/kg/day of LiCl *via* their drinking water for the entire HFD feeding period (12 weeks). For both chow-fed diet and HFD studies, body mass was recorded weekly, whereas water intakes were recorded and the water bottles refreshed twice weekly per mouse. In addition, food intake was recorded weekly per mouse.

A partial muscle-specific GSK3 KD (GSK3^mKD^) colony was generated by crossing GSK3 (α and β) floxed mice with HSA-Cre mice (Jackson Laboratories; catalog no.: 006149). The GSK3 floxed mice were cryorecovered by Jackson Laboratories using cyrorecovered sperm kindly donated to us by Dr Virginia Lee (University of Pennsylvania). Both GSK3 floxed mice and HSA-Cre mice were on a C57BL/6J background. The GSK3^mKD^ mice are heterozygous GSK3 floxed mice that heterozygously express HSA-Cre. The GSK3 floxed mice not expressing HAS-Cre (GSK3^floxed^) were used as controls. These mice were maintained on a standard chow diet and kept on a 12-h light:12-h dark cycle and had ad libitum access to food and water through the entirety of the study. At 3 to 6 months of age, the mice were euthanized and their soleus muscles were collected.

### DXA scan

A small animal DXA scanner (OsteoSys InSIGHT; Scintica) was used to noninvasively measure body composition in anesthetized mice (vaporized isoflurane 5% in O_2_) at 4 and 6 weeks into the lithium treatment. Percent lean and fat mass were calculated using total body mass; lean mass and body mass were used to normalize daily energy expenditure.

### Metabolic cages

For the chow portion of the study, a Promethion metabolic caging unit was used to measure oxygen consumption, respiratory exchange ratio, cage activity, and food and water intake during a 48-h period in three separate trials. The first trial at 4 weeks of treatment was used to acclimate the mice to the metabolic cages. The second and third trials occurred on the 6th and 11th week of treatment and were used for data acquisition. Data were obtained for light, dark, and combined (*i.e.*, daily) cycles.

### Glucose and insulin tolerance testing

IP glucose tolerance testing (GTT) and insulin tolerance testing (ITT) were performed on the mice for the purpose of measuring whole-body glucose tolerance and insulin sensitivity, respectively. Tests were performed 48 h apart, and mice had free access to their respective diets and treatments in between testing. For the GTT, animals were fasted for 6 h prior to the IP glucose injection (2 g/kg body weight). Blood samples were taken from the tail vein at 0, 15, 30, 45, 60, 90, and 120 min postinjection with the use of a hand-held glucometer (Freestyle Lite; Abbott). Similarly, for the ITT, concentrations of blood glucose were determined by sampling blood from the tail vein at 0, 15, 30, 45, 60, and 90 min following the IP insulin injection (0.75 U/kg) with the use of a hand-held glucometer. Plots of the average changes in plasma glucose over time were made for each group, and the average total area under the curve was calculated. Area under the curve is presented in mmol/l × time, and baseline values are set to X = 0.

### Tissue collection and homogenization

After mice were euthanized *via* exsanguination (under general anesthetic), WAT, BAT, and soleus muscles were collected. WAT samples were collected from the left- and right-side inguinal fat depots (iWAT), and BAT samples were collected from the interscapular fat pads. The samples were either placed into formalin (adipose) for histological analysis or snap frozen in liquid nitrogen and stored at −80 °C for SERCA functional analysis and Western blotting.

Adipose tissue samples were homogenized *via* FastPrep (MP Biomedicals) in 3× and 10× volume to weight for WAT and BAT samples, respectively, in cell lysis buffer (NP40 Cell Lysis Buffer [Life Technologies; catalog no.: FNN0021]) supplemented with 34 μl PMSF and 50 μl protease inhibitor cocktail (Sigma; catalog nos.: 7626-5G and P274-1BIL). Homogenates were centrifuged at 4 °C for 10 min at 1500*g* after which the supernatant was collected. Soleus muscle samples were homogenized in buffer containing 250 mM sucrose, 5 mM Hepes, 0.2 mM PMSF, and 0.2% (w/v) NaN_3_. Protein concentration of all homogenates was determined using a Bicinchoninic acid assay.

### Histology

Samples of iWAT and BAT were fixed in 10% neutral-buffered formalin (VWR; catalog no.: 16004-126) for 48 h and then transferred to 70% ethanol for future processing. Samples underwent dehydration *via* ethanol (1 × 90% 30 min, 3 × 100% 40 min) and xylene (Fischer Scientific) (3 × 45 min) and embedded in paraffin. About 10 micrometer sections were mounted on 1.2 mm Superfrost slides, stained with modified Harris H&E, and imaged at 20× magnification (BioTek Cytation 5). Three images from each animal (∼150 cells/image) were sampled to determine cross-sectional area and percent multilocular (ImageJ software; National Institute of Mental Health).

### SERCA coupling ratio

The apparent coupling ratio of SERCA was determined by combining a fluorescent Ca^2+^ uptake assay fitted onto a 96-well plate using the ratioable dye Indo-1 and a spectrophotometric SERCA activity assay that measures rates of ATP hydrolysis (6, 8). An M2 Molecular Device plate reader was used for both assays. Briefly, for Ca^2+^ uptake, muscle homogenates or cell lysates were plated in duplicate, and uptake was initiated upon the addition of ATP. Fluorescence of Ca^2+^-bound Indo-1 (405 nm emission) and Ca^2+^-free Indo-1 (485 nm emission) was measured upon excitation at 355 nm. The ratio of Ca^2+^-bound to Ca^2+^-free Indo-1 (405/485 nm) along with the known dissociation constant of 250 nM was used to calculate [Ca^2+^]_free_. SERCA coupling ratio was then calculated by dividing the rate of Ca^2+^ uptake by the rate of ATP hydrolysis (*i.e.*, SERCA activity).

### Western blotting

Glycine-based SDS-PAGE was used to electrophoretically separate solubilized proteins (in 1× Laemmli buffer) that were then transferred onto either nitrocellulose or polyvinylidene difluoride membranes. Membranes were probed for phosphorylated (serine 9) GSK3β (catalog no.: 9336, 1:1000 dilution; Cell Signalling), GSK3β (catalog no.: 9315, 1:1000 dilution; Cell Signalling), SERCA1a (catalog no.: MA3-912, 1:2000 dilution; Thermo Fisher Scientific), SERCA2a (catalog no.: MA3-919, 1:2000 dilution; Thermo Fisher Scientific), RYR1 (catalog no.: MA3-925, 1:1000 dilution; Thermo Fisher Scientific), RYR2 (catalog no.: PA5-87416, 1:1000 dilution; Thermo Fisher Scientific), NNAT (catalog no.:26905-1AP, 1:1000 dilution; Proteintech), SLN (catalog no.: ABT13, 1:200 dilution; Sigma–Aldrich), cytochrome C (catalog no.: ab76237, 1:1000 dilution; Abcam), citrate synthase (catalog no.: ab96600, 1:1000 dilution; Abcam), COXIV (catalog no.: ab16056, 1:1000 dilution; Abcam), PDH (catalog no.: AB52082, 1:1000 dilution; Millipore), UCP1 (catalog no.: ab10983, 1:1000 dilution; Abcam), and PGC-1α (catalog no.: AB3242, 1:1000 dilution; Millipore). Anti-rabbit (catalog no.: 711-035-152) and antimouse (catalog no.: 115-035-033) horseradish peroxidase–conjugated secondary antibodies (Jackson ImmunoResearch; 1:2000 ratio) were used, and protein signals were detected by enhanced chemiluminescence using Western Lightning Plus ECL (catalog no.: NEL103E001; PerkinElmer) or Immobilon substrate (catalog no.: WBULS0500; Millipore) and imaged using a ChemiDoc Imaging System (Bio-Rad). SLN content was detected using a Tricine-based gel (50) and SuperSignal FemtoWest substrate (catalog no.: 340966; Thermo Fisher Scientific). Absorbances were quantified using ImageLab (Bio-Rad) and normalized to Ponceau stain to ensure equal protein loading.

### Statistical analysis

For the chow-fed study and GSK3 KD study, most comparisons were made with a two-tailed Student’s *t* test, and for the HFD study, most comparisons were made using a one-way ANOVA and a Tukey’s post hoc test. Two-way repeated-measures ANOVAs were used for the analysis of body mass, cumulative food intake, body composition, GTT and ITT, with a Tukey’s (HFD study) or Bonferonni’s (chow study) post hoc test. Statistical significance was assumed at *p* ≤ 0.05, and GraphPad Prism 8 (GraphPad Software, Inc) software was used to perform all statistical analyses. Results are stated as mean ± SEM.

## Data availability

All data are contained within this article and supporting information. Data can be made available upon request of the corresponding authors.

## Conflict of interest

The authors declare that they have no conflicts of interest with the contents of this article.
